# Prognostic Prediction Using the Clinical Data and Ultrasomics-Based Model in Acute Respiratory Distress Syndrome (ARDS) Combined with Acute Kidney Injury (AKI)

**DOI:** 10.1155/2022/4822337

**Published:** 2022-05-11

**Authors:** Xing Cai, Jing Li, Ping Qin, Peng An, Hao Yang, MingYan Zuo, Jinsong Wang

**Affiliations:** ^1^Department of Respiratory Medicine, Xiangyang Hospital of Traditional Chinese Medicine, Xiangyang Central Hospital, Xiangyang, Hubei 441000, China; ^2^Department of Internal Medicine, Xiangyang Central Hospital, Affiliated Hospital of Hubei University of Arts and Science, Xiangyang, Hubei 441000, China; ^3^Department of Internal Medicine, Xiangyang No. 1 People's Hospital, Hubei University of Medicine, Xiangyang 441000, China; ^4^Department of Radiology, The Affiliated Hospital of Nanjing University of Chinese Medicine, Jiangsu Province Hospital of Chinese Medicine, The First Clinical Medical College, 155 Hanzhong Road, Nanjing 210029, Jiangsu, China; ^5^Department of Ultrasound, Taizhou Hospital of Traditional Chinese Medicine, Taizhou, Jiangsu 225300, China

## Abstract

**Objective:**

A model was constructed based on clinical and ultrasomics features to predict the prognosis of patients in the respiratory intensive unit (RICU) who had acute respiratory distress syndrome (ARDS) combined with acute kidney injury (AKI). AKI ensues after ARDS in RICU ordinarily. The prognostic prediction tool was further developed on this basis.

**Methods:**

We collected clinical and ultrasonic data from 145 patients who had ARDS combined with AKI and received continuous renal replacement therapy (CRRT) in the RICU of Xiangyang Hospital of Traditional Chinese Medicine from March 2016 to November 2019. The patients were divided into the survival group (*n* = 51) and the death group (*n* = 94), depending on the treatment outcome. The training set (*n* = 102) and the testing set (*n* = 43) were established based on patient data. The clinical and ultrasomics features and the CRRT parameters were compared between the two groups. The influence factors of death were analyzed by logistic regression, and four predictive models were established. The predictive performance of 4 models was compared using the *R* Software 4.1.3. The decision curve analysis graphs were drawn using the *R* language to determine the net benefit of each.

**Result:**

Univariate analysis was conducted in the training set. The following risk factors for poor prognosis were identified: age, concurrent cancers, sequential organ failure assessment score (SOFA), number of organ dysfunctions, positive cumulative fluid balance at 72 h, time from ICU admission to CRRT, mean arterial pressure, oxygenation index, and gray-level size zone matrix, GLSZM (SumEntropy.239/SmallDependenceHighGrayLevelEmphasis.314/Maximum.327/Variance.338) (*P* < 0.05). Four models were built based on the above factors: clinical model, CRRT model, ultrasomics-based model, and combination model. Comparison using the MedCalc software indicated that the best predictive performance achieved with the combination model. The decision curve analysis also suggested that the combination model had the highest net benefit. Similar results were reported after validation on the testing set.

**Conclusion:**

The prognosis of ARDS patients combined with AKI is usually poor. The combination model based on clinical and ultrasomics features had the highest predictive performance. This model can be used to improve the clinical outcome and prognosis.

## 1. Introduction

Acute respiratory distress syndrome (ARDS) is characterized by hypoxemia. The patients generally present with acute respiratory distress, pneumonedema, and refractory hypoxemia. ARDS can be life-threatening. The incidence of ARDS is reported to be 13% in the respiratory intensive unit (RICU). Acute kidney injury is common in ARDS patients. It has been reported that the incidence of AKI is 25%–60% in ARDS patients receiving mechanical ventilation [1, 2]. The patients' survival rate decreases substantially when AKI is combined with ARDS. The inpatient mortality is 68% in patients with the combined conditions vs. 28% in ARDS patients without AKI. Continuous renal replacement therapy (CRRT) is the most effective treatment for AKI combined with ARDS [2–4]. Multiple organ support therapy is now available with CRRT. Although CRRT can dramatically reduce the mortality of critically ill patients, some die despite CRRT. It is imperative to develop a prognostic prediction tool for ARDS combined with AKI. In this study, we discussed the clinical and ultrasomics features in ARDS patients who were combined with AKI and received CRRT. The factors affecting deaths were analyzed in these patients. We further developed several predictive models to inform the clinical treatment decisions.

## 2. Subjects and Methods

### 2.1. Subjects

**Figure 1:**
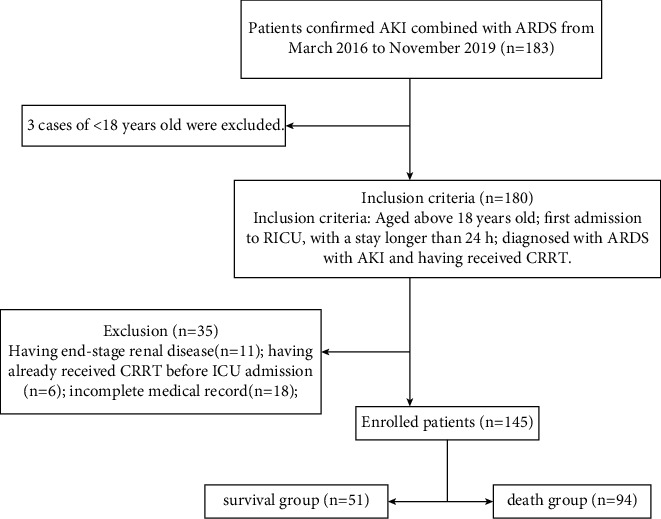
Flowchart showing inclusion and exclusion of subjects in this study.

We collected data from 145 ARDS patients who were combined with AKI and admitted to the RICU of Xiangyang Hospital of Traditional Chinese Medicine and Xiangyang Central Hospital from March 2016 to November 2019, and AKI occurrence time in RICU is 6.32 ± 2.35 days. Inclusion criteria: aged above 18 years old; first admission to RICU, with a stay longer than 24 h; and diagnosed with ARDS with AKI and having received CRRT. Exclusion criteria: having end-stage renal disease; having already received CRRT before ICU admission; and incomplete medical record. The patients were divided into the survival group (*n* = 51) and death group (*n* = 94), depending on the treatment outcome. Survival was defined as achieving stable disease and improvement and being discharged from ICU. The death group consisted of patients who died during RICU admission or subsequent follow-up after giving up treatment and being discharged against medical advice (Figure 1).

### 2.2. Clinical Data

Age, gender, body mass index (BMI), mean arterial pressure, RICU length of stay, complications, number of organ dysfunctions, blood gas analysis, oxygenation index, biochemical indicators, reasons to start CRRT, time from RICU admission to CRRT, CRRT duration, and intervention measures, such as mechanical ventilation and the use of vasoactive drugs, were collected. The worst indicator values were recorded within 24 h after RICU admission. These values were used to calculate the acute physiology and chronic health evaluation II (APACHE II) score and the sequential organ failure assessment (SOFA) score [5, 6].

### 2.3. CRRT Parameters

The Prismaflex bedside blood purification system with the filter was used for CRRT via femoral vein catheterization and internal jugular central venous catheterization. The CRRT mode was continuous venovenous hemofiltration or continuous venovenous hemodiafiltration. Citric acid or heparin was used as an anticoagulant in CRRT, or no heparin was used for CRRT [6, 7]. The dosage was 20–35m*∗*kg^−1^ *∗*h^−1^.

### 2.4. Ultrasomics

The Mindray R8 ultrasound scanner with the abdominal convex-array probe (frequency 3.0–5.5 MHz) was used. Bilateral renal cortex and parenchyma, renal collecting system, and ureters were scanned conventionally, and the images were stored. 3D Slicer version 4.11.20210226 (https://www.slicer.org/) was employed for ROI delineation on the images. Texture analysis and data extraction were performed. The first-order, GLCM, shape, and NGTDM features extracted from the images were analyzed by Lasso regression using *R* programming (version 4.1.3; https://www.r-project.org/). A valid set of texture features was extracted [8].

#### 2.4.1. Notes

(1) First-order: the first-order statistics describe the distribution of voxel intensity in the image region through common and basic measures

(2) GLCM: GLCM captures/refers to the spatial relationship of pixel pairs or voxel pairs with predefined gray intensity in different directions (horizontal, vertical, or diagonal of 2D analysis or 13 directions of 3D analysis) and predefined distances between pixels or voxels.

(3) Shape: shape refers to the measurement of mesh volume/area/perimeter, which is calculated from the triangular mesh of image ROI

(4) NGTDM: NGTDM refers to the sum of the differences between the gray level of quantized pixels or voxels and the average gray level of adjacent pixels or voxels within a predefined distance.

### 2.5. Statistical Methods

All statistical of mechanical analyses were performed using the *R* version 4.1.3 (*R* Foundation for Statistical Computing, version 4.1.3; https://www.r-project. org/). Measurement data obeying a normal distribution were represented by X ± s, and intergroup comparisons were performed by using the independent-samples *t*-test or by the *χ*^2^ test or Fisher's exact test. Multiple logistics regression analysis was carried out. The odds ratio (OR) and the 95% confidence interval (95% CI) were calculated, based on which the correlation between the risk factors and the prognosis was assessed. *P* < 0.05 was taken to indicate a significant difference. Receiver operating characteristics (ROC) curves were generated and the area under the curve (AUC) was used to evaluate the accuracy of 4 models in prognostic prediction. Then, the developed models were validated by assessing their prediction performance in the test set. Comparisons between 4 models were performed using the DeLong test in the training and test sets. Higher prediction accuracy presented with a larger AUC and a *P* value <0.05 (two-tailed) indicated statistical significance. Decision curve analysis (DCA) of training and test sets were conducted to determine the clinical usefulness by quantifying the net benefits at different threshold probabilities in the models [9].

## 3. Results

### 3.1. General Information

Univariate logistic regression showed that age, concurrent cancers, SOFA score, mean arterial pressure, number of organ dysfunctions, positive cumulative fluid balance at 72 h, time from ICU admission to CRRT, and oxygenation index were risk factors for death after CRRT in ARDS patients combined with AKI (*P* < 0.05) (Table 1 and Table 2).

### 3.2. Ultrasomics

**Figure 2:**
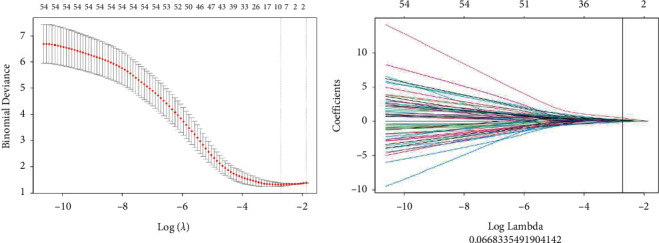
Schematic diagram of texture omics feature extraction based on *R* Studio software (Lasso regression method). A total of 11 groups of available texture data are extracted.

Our team extracted 874 groups of texture data from the regions of interest in the two kidneys using the 3D Slicer software. Lasso regression in R programming identified eleven valid groups of texture data. Next, the normal distribution test and independent-samples *t*-test were carried out. Four valid groups of data were finally selected, that is, the gray-level size zone matrix (Sum Entropy.239/Small Dependence High Gray Level Emphasis.314/Maximum.327/Variance.338) (Table 3 and Figure 2).

**Figure 3:**
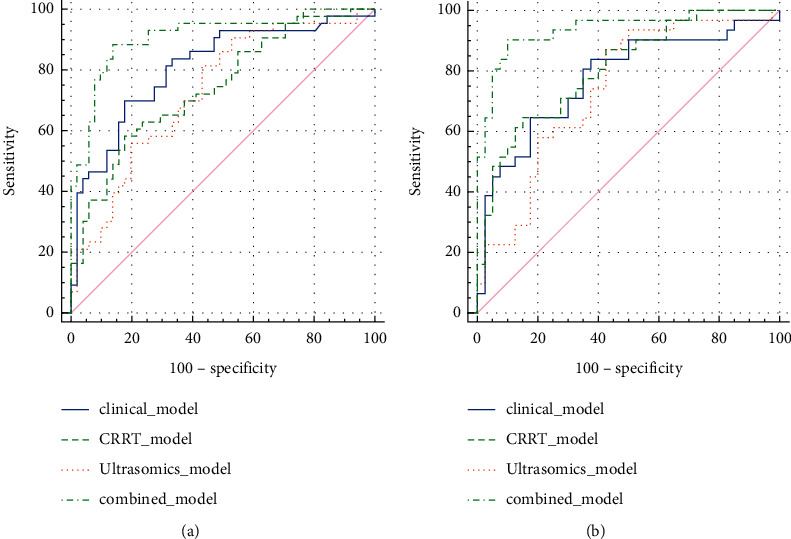
DeLong nonparametric method was used to estimate the area under the curve of ROC between different prediction models of training set (a) and test set (b) and compare its effectiveness in predicting the prognosis of ARDS and AKI. The area under the curve of the combined model was the largest.

**Figure 4:**
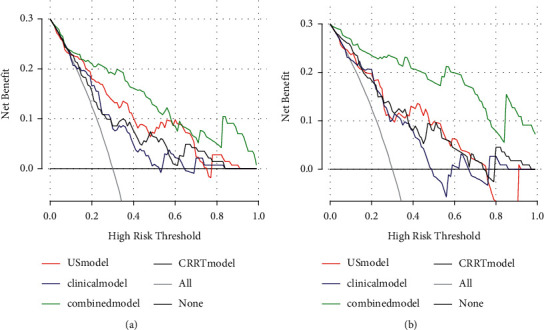
In the training set (a) and the test set (b), the prediction performances of the ultrasomics model, clinical model, CRRT model, and combined model are compared using the net benefit of decision curve; it is confirmed that the combined model had the highest predictive performance.

Four predictive models were built on the training set consisting of data on the above risk factors (clinical model, CRRT model, ultrasomics-based model, and combination model). The predictive performance of the four models was compared using the MedCalc v20.0.22 software. The combination model had the highest predictive value on the training set (AUC: 0.910 (OR 0.0315, 95% CI: 0.848–0.972)). Its predictive performance was much higher than that of the clinical model (AUC: 0.801 (OR 0.0461, 95% CI: 0.719–0.899), *P*=0.0132), ultrasomics-based model (AUC: 0.741 (OR 0.0511, 95%CI: 0.641–0.842), *P*=0.0003), and CRRT model (AUC: 0.728 (OR 0.0522, 95% CI: 0.626–0.831), *P*=0.0004). The decision curve analysis graphs plotted using R programming confirmed that the combination model had a considerably higher net benefit. This result was later validated on the testing set, that is, the combination model had the highest predictive performance (AUC: 0.938 (OR 0.0298, 95% CI: 0.879–0.996)). The difference was significant compared with the clinical model (AUC: 0.776 (OR 0.0583, 95% CI: 0.661–0.891), *P*=0.0013), ultrasomics-based model (AUC: 0.812 (OR 0.0509, 95% CI: 0.710–0.909), *P*=0.0013), and CRRT model (AUC: 0.741 (OR 0.0595, 95% CI: 0.624–0.857), *P*=0.0009) (Figure 3 and Figure 4).

## 4. Discussion

There has been an increasing number of studies on kidney-lung interactions in recent years. Acute lung injury is considered the result of kidney-lung interactions in AKI, while AKI is the result of kidney-lung interactions in acute respiratory failure. In ARDS patients, mechanical ventilation may induce kidney injury by causing hemodynamic abnormalities and biological trauma or via the neurohormonal effect. ARDS may also induce kidney injury by inducing gas exchange disorder, inflammatory response, and oxidative stress. Lung injury is a common condition associated with AKI, primarily through the following pathways: body fluid overload, increased pulmonary vascular permeability, cytotoxicity, and oxidative stress. ARDS combined with AKI, a condition common in critically ill patients, is difficult to treat [10, 11].

We compared the baseline parameters between the survival group and the death group and found no significant differences in gender, BMI, reasons for ICU admission, and causes of ARDS. However, patients in the death group were older in age than in the survival group. The percentage of patients with concurrent cancers was also higher in the death group. The organ reserves and compensation decrease significantly with age. Elderly patients are usually combined with various chronic and underlying diseases, which makes the patients' conditions even worse and precarious. Those with concurrent cancers are more vulnerable to other diseases due to their poor overall health status [12, 13]. In the present study, the SOFA score, number of organ dysfunctions, and the positive cumulative fluid balance at 72 h were significantly higher in the death group than in the survival group. However, the oxygenation index and the mean arterial pressure were much lower in the death group than in the survival group. The SOFA score allows for calculating both the number and the severity of organ dysfunction in six organ systems. It is a convincing and widely used indicator of organ dysfunction severity and also a prognostic predictor. Multiple organ dysfunction syndrome is considered a risk factor for death in ICU patients [14, 15]. A low mean arterial pressure usually indicates poor blood perfusion and probably more severe injury to the heart, brain, and kidney. Fluid overload can cause severe injury to critically ill patients. It has been reported that positive cumulative fluid balance is related to the mortality of ICU patients. The oxygenation index is a comprehensive indicator of pulmonary oxygenation state. It can reflect the hypoxic state, pulmonary blood flow, and alveolar gas exchange and predict the outcome [16–18].

CRRT is a blood purification therapy for salvaging critically ill patients and has undergone dramatic development in recent years. This therapy has been widely applied to emergency patients with multiple organ dysfunction syndrome and critical AKI. ARDS patients with AKI suffer from the systemic inflammatory response to varying degrees [18, 19]. CRRT can help remove excess fluid, metabolites, and inflammatory mediators, thereby relieving systemic inflammatory response, improving the immune function, and fastly regulating the acid-base balance and electrolyte balance. Besides, CRRT can maintain fluid balance and homeostasis, redressing the symptoms of shock. CRRT is critical for improving the prognosis of critically ill patients [20–22]. We found that the timing of CRRT was equally important, and early CRRT could dramatically reduce the death risk for patients.

The concept of radiomics was first proposed by Holland researcher Lambin in 2012. Radiomics was initially applied to X-rays. It was soon found that computer vision outperformed the naked eyes in X-ray image processing. 3D Slicer and ITK-SNAP are softwares jointly developed by several universities, including Harvard University. With the aid of the software, omics has been extended to several examination modalities, including ultrasound, CT, MRI, and pathology. In the past decade, high-IF, high-quality radiomics studies have appeared extensively. We used radiomics parameters to study ARDS combined with AKI, achieving a high benefit. We delineated the regions of interest on the ultrasound images from 145 patients. After the software-assisted screening, we extracted four valid groups of ultrasomics features, representing the signal intensity, density, and grayscale of the lesions. We built four predictive models based on the abovementioned risk factors, which were trained and validated on the training set and the testing set, respectively. The findings were consistent on the training and testing sets. Finally, the DeLong nonparametric test and decision curve analysis confirmed that the combination model had the highest predictive performance, its AUC being significantly higher than that of other models. This was probably because the combination model integrated the clinical and ultrasomics features and estimated parameters representing interindividual differences in ARDS patients with AKI. As a result, the predictive accuracy of the model was increased. The prognostic predictive model can predict the prognosis patients at an early stage, guide the intervention regimen for individuals, reduce malignant progression of diseases, and improve the survival quality and survival rate of critically ill patients [23–25].

### 4.1. Limitations

Predictive analytics have been widely used in the literature with respect to clinical research and risk stratification. However, most predictive analytics in this field exploit generalized linear models for predictive purposes, which are limited by model assumptions, including linearity between response variables and additive interactions between variables. In many instances, such assumptions may not hold true, and the complex relationship between predictors and response variables is usually unknown. To address this limitation, we will model the ARDS-AKI underlying data using machine-learning algorithms in future, which usually do not require strict assumptions regarding data structure, and they are able to learn complex functional forms using a nonparametric approach. Furthermore, two or more machine-learning algorithms (ensemble modeling) can be synthesized to further improve predictive accuracy. In addition, the data size was small; selective bias was inevitable for a single-center study. CT-MRI radiomics study was not included [26, 27].

## 5. Conclusions

To conclude, the mortality remained high after CRRT in ARDS patients combined with AKI. Concurrent cancers, number of organ dysfunctions, oxygenation index, time from ICU admission to CRRT, and gray-level size zone matrix were independent risk factors for death. The combination model built based on the above factors had the best prognostic prediction performance for ARDS patients combined with AKI. The model offers support for developing timely prevention and treatment measures for such patients.

## Figures and Tables

**Table 1:** Regression analysis results of establishing the clinical model based on clinical characteristics to predict the prognosis of ARDS and AKI.

Clinical characteristics model	Univariate analysis		Multivariate analysis	
	*P*	Hazard ratio	*P*	Hazard ratio
Age	0.015^*∗*^	1.076 (1.014–1.141)		
Gender	0.772	0.894 (0.956–1.034)		
BMI	0.069	0.631 (1.021–2.003)		
Concurrent cancers	0.002^*∗*^	1.906 (1.277–2.844)	0.002^*∗*^	2.021 (1.302–3.135)
ICU length of stay	0.553	0.239 (1.071–1.659)		
APACHE II	0.312	0.979 (0.939–1.020)		
SOFA score	0.016^*∗*^	1.078 (1.015–1.146)		
Mean arterial pressure	0.017^*∗*^	1.033 (1.006–1.061)		
Mechanical ventilation time	0.341	0.851 (1.075–2.701)		
Number of organ dysfunctions	0.014^*∗*^	1.131 (1.025–1.249)	0.035^*∗*^	1.125 (1.008–1.256)
Leukocyte count	0.395	0.788 (0.596–1.788)		
Hemoglobin	0.431	0.683 (0.998–1.518)		
AST	0.631	0.758 (0.673–0.968)		
ALT	0.839	0.331 (0.083–1.701)		
Albumin	0.341	0.851 (0.938–1.331)		
Creatinine	0.553	0.639 (1.231–1.159)		
Carbon dioxide tension	0.639	0.839 (1.236–2.177)		
Oxygen partial pressure	0.753	0.935 (1.535–1.938)		
Positive cumulative fluid balance at 72 h	0.032^*∗*^	1.019 (1.002–1.036)		
AKI occurrence time in RICU	0.954	0.995 (0.849–1.167)		

^*∗*^ *P* < 0.05.

**Table 2:** Regression analysis results of establishing the CRRT model based on CRRT parameters to predict the prognosis of ARDS and AKI.

CRRT model	Univariate analysis		Multivariate analysis	
	*P*	Hazard ratio	*P*	Hazard ratio
Reasons for CRRT	0.669	1.012 (0.935–1.133)		
CRRT vascular pathway	0.821	0.881 (0.856–1.002)		
Treatment mode (CVVH/CVVHDF)	0.159	0.935 (0.697–1.003)		
Anticoagulant therapy	0.622	0.782 (0.717–1.227)		
Therapeutic dose	0.312	0.811 (1.321–1.699)		
Time from ICU admission to CRRT	0.002^*∗*^	1.191 (1.066–1.332)	0.002^*∗*^	1.202 (1.071–1.351)
CRRT duration	0.141	0.891 (1.212–1.642)		
Oxygenation index	0.031^*∗*^	1.026 (1.002–1.051)	0.025^*∗*^	1.028 (1.003–1.053)

^*∗*^ *P* < 0.05.

**Table 3:** Regression analysis results of establishing the ultrasomics model based on ultrasomics parameters to predict the prognosis of ARDS and AKI.

Ultrasomics model	Univariate analysis		Multivariate analysis	
	*P*	Hazard ratio	*P*	Hazard ratio
Difference Variance.103	0.797	0.969 (0.762–1.232)		
Small Dependence Emphasis.130	0.593	1.076 (0.822–1.411)		
Difference Average.223	0.120	1.276 (0.938–1.735)		
Sum Entropy.239	0.042^*∗*^	1.024 (1.001–1.048)	0.025^*∗*^	1.029 (1.004–1.055)
Small Dependence High Gray Level Emphasis.314	0.035^*∗*^	0.982 (0.966–0.988)		
Maximum.327	0.021^*∗*^	0.976 (0.956–0.996)	0.028^*∗*^	0.976 (0.954–0.997)
Variance.338	0.034^*∗*^	1.013 (1.001–1.025)	0.034^*∗*^	1.014 (1.001–1.026)
Idm.349	0.793	1.017 (0.896–1.154)		
Small Area High Gray Level Emphasis.665	0.059	1.300 (0.990–1.707)		
Run Variance.755	0.317	0.648 (0.777–1.516)		
Strength.872	0.484	1.237 (0.682–2.242)		

^*∗*^ *P* < 0.05.

## Data Availability

The data generated or analyzed during this study are included within this article.
